# The influence of different hinge position on PTS during HTO: comparison between open-wedge and closed-wedge HTO

**DOI:** 10.1007/s00590-022-03280-5

**Published:** 2022-05-31

**Authors:** Dong-Kyu Moon, Min-Seok Seo, Chang-Won Kim, Seong-Hee Cho, Dae-Cheol Nam, June-Ho Byun, Sun-Chul Hwang

**Affiliations:** 1 Department of Orthopaedic Surgery, Institute of Health Science, Research Institute of Life Science, and Collage of Medicine, Gyeongsang National University, Jinju, Republic of Korea; 2 Department of Oral and Maxillofacial Surgery, Institute of Health Science, Research Institute of Life Science, and Collage of Medicine, Gyeongsang National University, Jinju, Republic of Korea

**Keywords:** Open-wedge high tibial osteotomy, Closed-wedge high tibial osteotomy, Hinge position, Posterior tibial slope, Medial proximal tibial angle

## Abstract

**Purpose:**

The purpose of this study was to determine the significance of hinge position through comparison between open-wedge and closed-wedge high tibial osteotomy (HTO) and to determine the ideal hinge position to minimize the effect of HTO on the posterior tibial slope (PTS) and medial proximal tibial angle (MPTA).

**Methods:**

Procedures were performed on 32 cadaveric knees using open-wedge HTO with the standard hinge position or a low hinge position or closed-wedge HTO with the standard hinge position or a low hinge position. To define the standard hinge position in open wedge HTO, we drew a line 3-cm inferior to the medial tibial plateau toward the fibular head and located the intersection of this line with a longitudinal line 1-cm medial to fibular shaft. The low hinge position was then defined as the point 1-cm inferior to the standard position. For the standard hinge position for closed-wedge HTO, we drew a line parallel with joint line from 2-cm inferior to the lateral tibial plateau. The low hinge position was then defined as the point 1-cm inferior to the standard position.

**Results:**

For the open-wedge procedure, osteotomy through the low hinge position resulted in a significantly greater PTS compared to osteotomy through the standard hinge position. MPTA was also significantly greater for the low hinge position compared to standard hinge position. In the closed-wedge HTO, neither the PTS nor MPTA was significantly different for the low and standard hinge positions.

**Conclusions:**

Hinge position significantly affects changes in the PTS and MPTA following open-wedge but not closed-wedge HTO. Understanding how to hinge position affects the PTS and MPTA is critical for surgeons performing open-wedge HTO procedures. Adopting an accurate hinge position is crucial for preventing complications, especially in open-wedge osteotomy, due to postoperative changes in the PTS and MPTA.

## Introduction

Knee osteoarthritis (OA) is a joint dysfunction due to degeneration, destruction, and loss of articular cartilage. Varus malalignment applies excessive stress on the medial tibiofemoral compartment, causing degenerative changes, which lead to pain and dysfunction of knee joint [1, 2]. Surgical treatment may include high tibial osteotomy (HTO) or arthroplasty; however, there is still controversy over the optimal surgical indication for younger patients in many aspects [3]. Considerable surgical options for younger and active patients include HTO and unicompartmental knee arthroplasty (UKA). HTO is comparatively indicated in young and more active patients [4, 5]. HTO is a procedure that transfers the mechanical axis so that it is located further toward the center of the knee. As a result, this surgery improves pain and delays arthritis progression [6, 7].

There are two techniques for HTO for varus malalignment: medial open-wedge and lateral closed-wedge HTO. Open-wedge HTO offers the advantages of easier surgical techniques and less risk of neurovascular injury [8–12], but it has a risk of unintentional alteration of the posterior tibial slope (PTS). Many studies reported that the PTS increased after open-wedge HTO. On the other hand, closed-wedge HTO offers advantages of accelerated healing of the bone due to the positioning of the osteotomy in the metaphysis, but this technique also unintentionally alters the PTS. Many studies reported that the PTS decreased after closed-wedge HTO [11, 13–19]. Previous studies have examined the biomechanical causes of decreased PTS following closed-wedge HTO and increased PTS following open-wedge, as well as the magnitude of PTS change following each technique. However, studies on PTS change according to hinge position are rare.

The purpose of this cadaveric study was to investigate how a different hinge position would affect PTS in medial open-wedge and lateral closed-wedge HTO. We hypothesized that a low hinge position would result in a greater increase and decrease in the PTS in open-wedge and closed-wedge HTO, respectively, compared to the standard position.

## Materials and methods

Thirty-two cadaveric knees (16 matched pairs) without a history of surgery, gross limb deformity, anterior cruciate ligament (ACL) or posterior cruciate ligament (PCL) injury, or notch stenosis were used. Matched pairs were used to minimize anatomical difference between specimens. The subjects included 11 males and 5 females. The demographic characteristics included subject age, sex, size, weight, height, body mass index (BMI), and tibial length. The median donor age was 64.3 years (43–82 years), median weight and height were 75.0 kg (45.0–88.0 kg) and 167.7 cm (150.0–180.0 cm), respectively, and median BMI was 26.9 kg/m^2^ (18.9–32.3 kg/m^2^). Detailed demographic characteristics are in Table 1.

**Table 1:** Demographic characteristics

Characteristic	Value
Age, yr	64.3 (43–82)
Sex, n	11 males and 5 females
Side, n	16 right and 16 left
Tibia length, cm	47.5 (35.2–53.5)
Weight, kg	75.0 (45.0–88.0)
Height, cm	167.7 (150.0–180.0)
Body mass index	26.9 (18.9–32.3)

Data are presented as mean (range) unless otherwise indicated

During the experimental HTO procedure, the proximal femur was clamped in a vice-grip construction. To confirm accurate hinge position and osteotomy entry site when performing HTO, we used cadaveric knee models with their soft tissue dissected. The standard hinge position was determined according to the most commonly described method reported by several previous studies [14–16, 18–20].

**Fig. 1:**
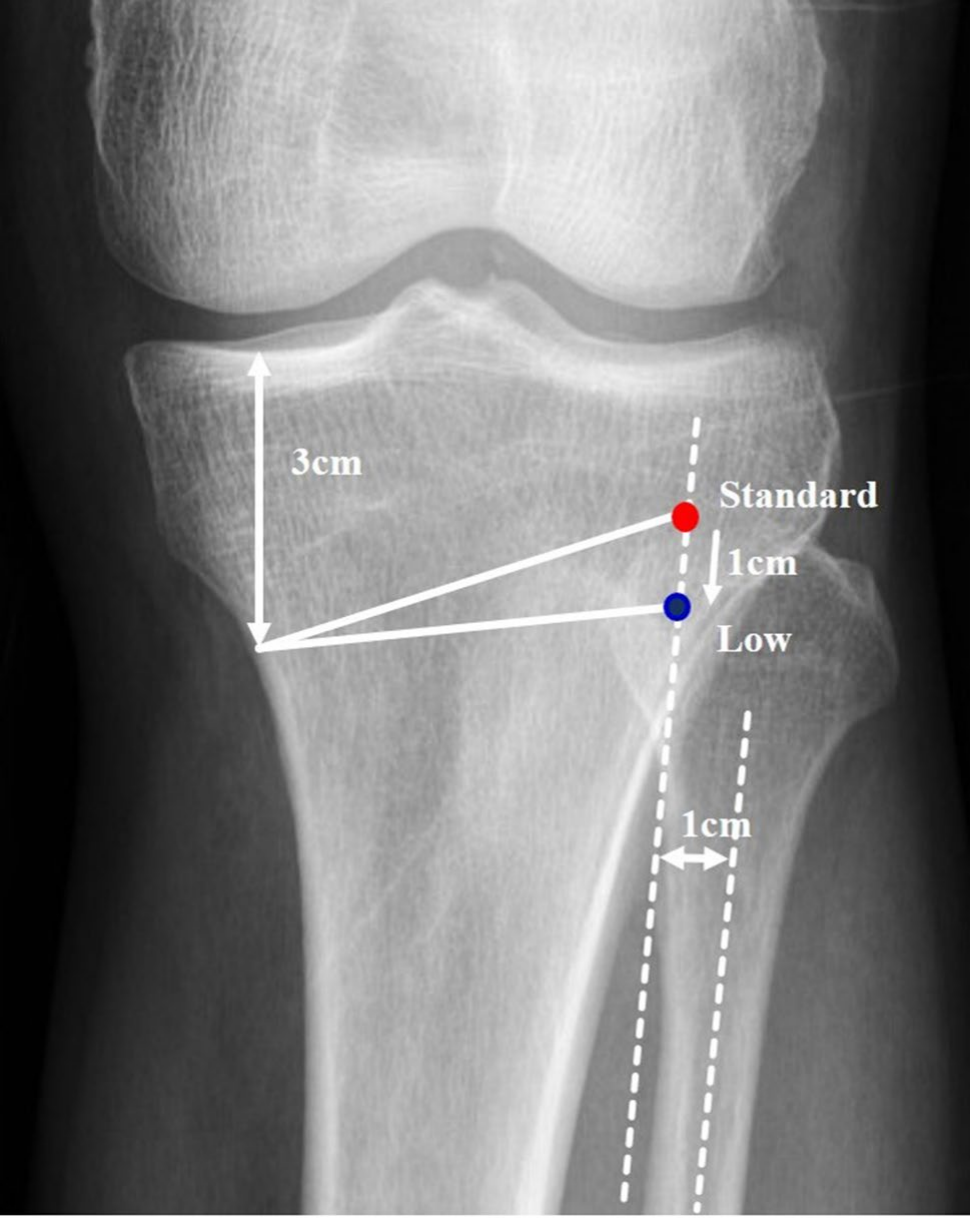
Hinge position placement of open-wedge HTO. Standard hinge position (red dot): the line was drawn toward the fibular head 3 cm inferior to the medial tibial plateau to intersect a longitudinal line 1 cm medial to the fibular shaft. Low hinge position (blue dot): 1 cm lower than standard hinge position. Osteotomy was performed based on the white line for each hinge position

In open-wedge HTO, the fibular head 3 cm inferior to the medial tibial plateau was made to cross with a longitudinal line 1 cm medial to the fibular shaft. Low hinge position was defined as 1 cm inferior to the standard position (Fig. 1). Open-wedge biplanar osteotomy was performed in all specimens using the Lobenhoffer surgical technique[21]. The tibial osteotomy was performed from the medial to the lateral side, with the most lateral 10% of the tibia kept intact. Great care was taken not to break through the lateral side, and the osteotomy site was slowly opened medially, with the proximal fragment evenly elevated in both the front and back. First, the two hinge positions were made, and then two 1.6 guide wires were inserted. One wire was inserted along the line toward the fibular head from 3 cm inferior to the medial tibial plateau. The other wire was positioned 1 cm below the intersection between the first wire and tibial medial border. The guide wire position was observed through the incision site. Next, the guide wires were located for the two hinge positions, and a saw was passed over the guide wires. Following this, all knees underwent biplane open-wedge tibial tuberosity osteotomy with a lateral cortical hinge, as described in a previous study that focused on preventing an unintended increase in PTS [14, 22]. The superficial medial collateral ligament was located at the osteotomy site and was cut along the planned osteotomy line. All knees were corrected by 10 mm at the medial tibial side [23, 24]. Eight cases had osteotomies through the standard hinge position, and the other eight cases had osteotomies through the low hinge position performed by a senior author.

**Fig. 2:**
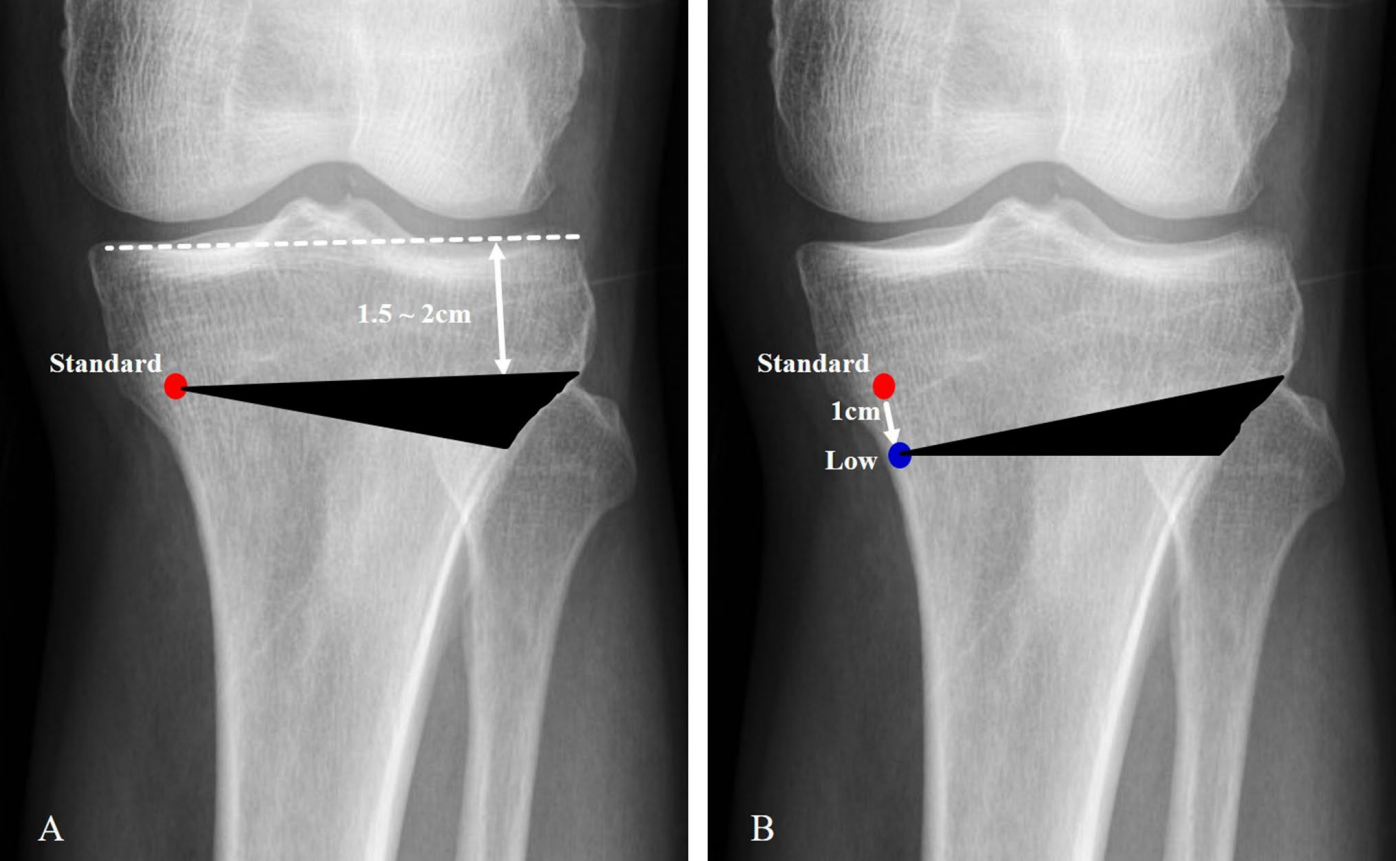
Hinge position placement of closed-wedge HTO. **A** Standard hinge position (red dot): the line parallel with joint line from 1.5 to 2 cm inferior to the lateral tibial plateau, **B** low hinge position (blue dot): 1 cm lower than standard hinge position. The wedge marked in black was removed during closed wedge high tibial osteotomy

In closed-wedge HTO, we drew a line parallel with joint line from 1.5 to 2 cm inferior to the lateral tibial plateau. The low hinge position was defined as 1 cm inferior to the standard position (Fig. 2). The first guide wire was inserted parallel to articular plane from lateral to medial side, and the second guide wire was inserted from 1 cm inferior to the first guide wire to the virtual triangle apex. Osteotomy was performed along the first and second guide wires. The apex was not osteotomed, and inner cortical bone was spared. Inner cancellous bone was set to act as a hinge, and two-thirds of the wedge including outer cortical bone was removed. Following wedge removal by varus force, the two sides of the osteotomy were in contact with each other and fixed with staples. Eight cases had osteotomies through the standard hinge position, and the other eight cases had osteotomies through the low hinge position performed by a senior author.

**Fig. 3:**
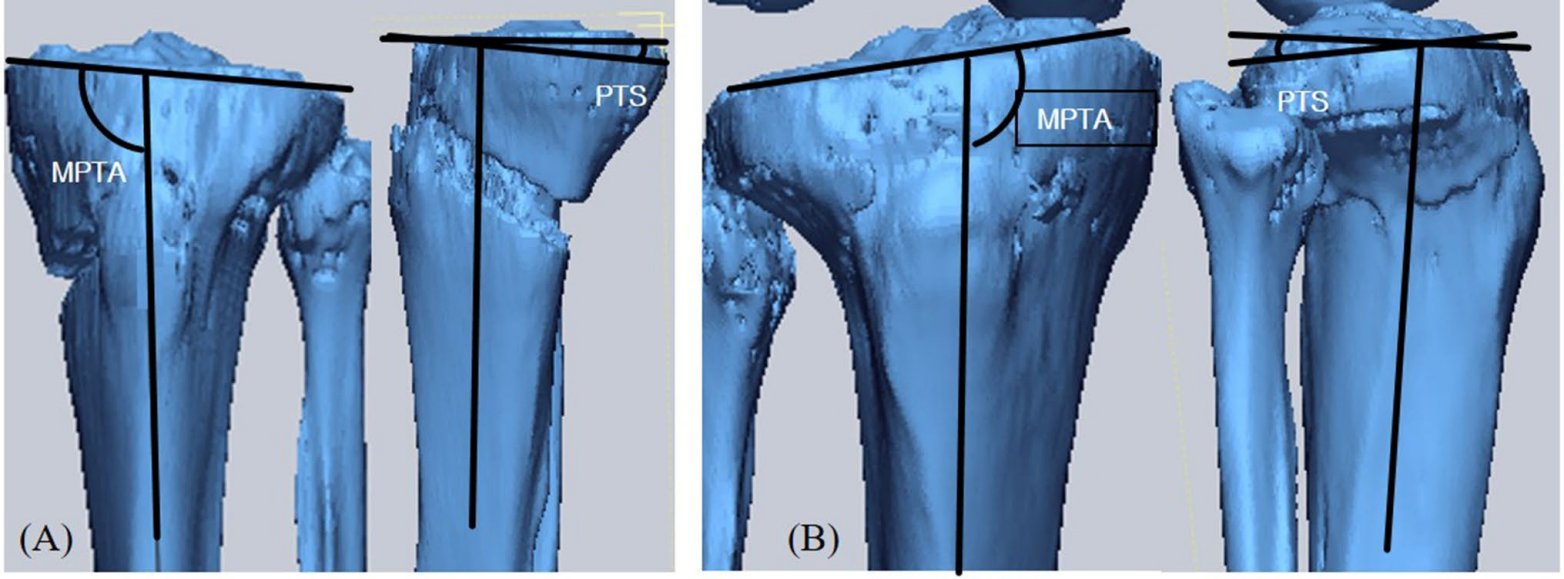
Measurement method for Posterior tibial slope (PTS) and medial proximal tibial angle (MPTA) from 3-dimensional computed tomography images. **A** Open-wedge HTO, **B** Closed-wedge HTO

After the osteotomies, computed tomography (CT) scans of each knee were performed. Bone morphology landmarks on CT were measured to determine the PTS and MPTA for each cadaveric knee. Using axial sections from the CT scans, the lateral tibial cortex was identified as compromised. A three-dimensional (3D) model of each knee was reconstructed using MIMICS® software (Mimics 12.3, Materialise, Leuven, Belgium) to identify the tibial hinge position orientation. Geomagic® Software (Research Triangle Park, North Carolina, USA) was used to create the virtual hinge position and perform 3D measurements. The 3D model was rotated sagittally until the best-fit plane was aligned on the 2-dimensional (2D) projected plane. This created the true anteroposterior (AP) view. After the true AP view was captured, the 3D model was rotated 90 degrees axially. The true lateral view was then completely captured. The captured true AP and lateral view were imported into ImageJ software (version 1.53q; National Institutes of Health, Bethesda, MD). Then, the PTS and MPTA were measured. The PTS was measured as the angle formed at the intersection of a line parallel to the posterior tibial inclination and a line that bisects the diaphysis of the tibia in the captured true lateral view. The MPTA was measured in the true AP view. The medial angle between the center line of the proximal tibia and the articular line of the tibial plateau formed the MPTA (Fig. 3). Two experienced orthopedic surgeons independently evaluated all measurements. Parameters were measured twice, with an interval of 2 weeks between measurements by each surgeon. The inter- and intra-observer reliabilities were satisfactory with mean values of 0.97 and 0.94, respectively.

### Statistical analyses

Power analysis indicated that a sample size of eight subjects per group would provide 80% statistical power to detect effect size (*d* = 0.66) between the groups (alpha = 0.05, beta = 0.20). SAS software (version 9.3; SAS Institute, Cary, NC) was used for statistical analyses. A *p*-value < 0.05 was considered statistically significant. A paired t-test was used to compare the PTS and MPTA between the standard and low hinge positions. Measurement reliability was assessed by examining the intraclass correlation coefficient. The Mann–Whitney test was used to compare bone morphometry of the cadaveric knees.

## Results

Demographic characteristics included subject age, sex, size, weight, height, BMI, and tibial length. There were no significant differences in demographic data between the two groups. Furthermore, there were no significant differences in the PTS or MPTA between subjects before osteotomy.

**Fig. 4:**
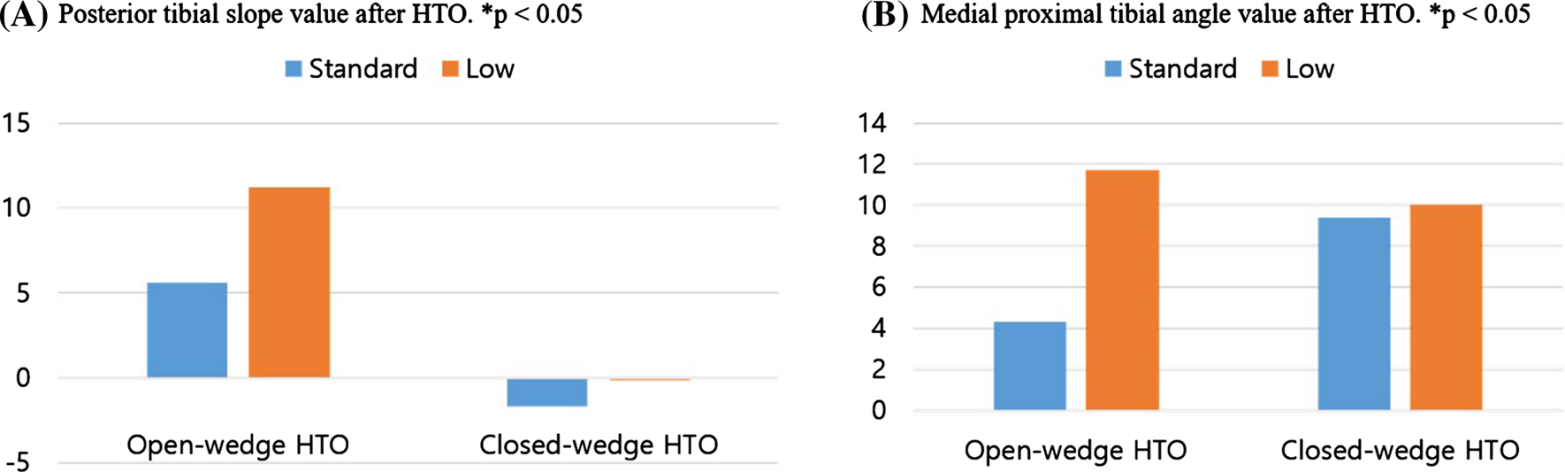
**A** Posterior tibial slope (PTS) and **B** medial proximal tibial angle (MPTA) values after HTO. *p < 0.05

In open-wedge HTO, the increase in PTS in the low hinge position group was significantly greater than in the standard hinge position group (standard: 5.6 ± 2.5° vs. low: 11.2 ± 3.0°, *p* < 0.001, Table 2). The mean MPTA of the low hinge position group was significantly increased compared to the standard position (standard: 4.3 ± 1.2° vs. low: 11.7 ± 1.6°, *p* < 0.001, Table 3) (Fig. 4). Lateral tibial cortex hinge fracture occurred in 2 of the 16 cases, both in the low hinge position group. Thus, despite the use of a safe zone, lateral tibial cortex fracture may be associated with the low hinge position technique (not significant).

**Table 2:** Posterior tibial slope measurements

PTS (°)	Standard hinge position (*n* = 8)	Low hinge position (*n* = 8)	*p*
Open-wedge HTO	5.6 ± 2.5	11.22 ± 3.0	< 0.001
Closed-wedge HTO	−1.7 ± 8.0	−0.13 ± 5.8	> 0.05

**Table 3:** Medial proximal tibial angle measurements

MPTA (°)	Standard hinge position (*n* = 8)	Low hinge position (*n* = 8)	*p*
Open-wedge HTO	4.3 ± 1.2	11.7 ± 1.6	< 0.001
Closed-wedge HTO	9.4 ± 4.3	10.0 ± 2.8	> 0.05

In closed-wedge HTO, osteotomy through the low hinge position resulted in no significant change compared to osteotomy through the standard hinge position in the PTS (standard: − 1.7 ± 8.0° vs. low: − 0.13 ± 5.8°, *p* > 0.05) or MPTA (standard: 9.42 ± 4.3° vs. low 10.0 ± 2.8°, *p* > 0.05) (Fig. 4).

## Discussion

The most important finding of this study was that a low hinge position during HTO resulted in a significantly greater increase in the PTS of open-wedge HTO than standard hinge position. There was no significant difference in the PTS for closed-wedge HTO in terms of hinge position. Our hypotheses were that tibial osteotomy in a low hinge position would cause abnormal osteotomy angles and that the low hinge position would be associated with greater PTS and decreased PTS of closed-wedge HTO. The results of this study support the hypothesis that the low hinge position during open-wedge HTO is significantly associated with malalignment, in turn, it could contribute to the subsequent mechanical failure after open-wedge HTO. Most previous investigations evaluated 2D pre- and postoperative radiographs, and these studies had limitations evaluating significant factors that influence the change in PTS. We employed 3D imaging to more fully characterize the effects of hinge position on HTO outcomes. The results of this study are clinically meaningful in that such 3D imaging increases the reliability of the results.

Based on a systematic review of open-wedge HTO studies, the technical factors of open-wedge HTO associated with its prognosis are not fully established. Among various surgical technical factors of medial open-wedge HTO, hinge position is considered one of the most important factor [14, 25, 26]. In general, the standard hinge position for open-wedge HTO is set along a line toward the fibular head 3 cm inferior to the medial tibial plateau and crossing a longitudinal line 1 cm medial to the fibular shaft. The standard hinge position for closed-wedge HTO is set along a parallel line with the joint line from 2 cm inferior to the lateral tibial plateau. Despite the importance of hinge position, and there are only few studies on the effect of hinge position on the PTS in closed-wedge HTO. The purpose of this study was to evaluate the effects of tibial hinge position (standard and low hinges) on outcomes for open-wedge and closed-wedge HTO, and such scarcity of reports on the issue strengthens this study.

An important finding in this study was that PTS further increased in medial open-wedge HTO and no significant difference in closed-wedge HTO when osteotomy was performed through the low hinge position. We compared eight standard tibial osteotomy hinge positions with eight low hinge positions in each group. In open-wedge HTO, the mean orientation angle for the PTS in the low hinge position group was 11.2 ± 3.0°. This angle represents a greater increase in the PTS compared to the mean orientation angle for the PTS for the standard hinge position group which was 5.6 ± 2.5°, and it contributed to greater MPTA. In addition, two of eight cases with low hinge position had lateral tibial cortex hinge fractures. This also suggests that in the low position, the improperly oriented angle shortens the tibial osteotomy length and, thereby, may contribute to surgical complications such as lateral tibial cortex hinge fracture. In closed-wedge HTO, the mean orientation angle for the PTS in the low hinge position group was −0.13 ± 5.8°. This angle in the PTS was not significantly different from that in the standard hinge position group, which was − 1.7 ± 8.0°.

Erik et al. [19] demonstrated that tibial slope change does not need to be evaluated with radiography when performing a closed-wedge HTO. Wang et al. [15] reported that a posterolateral position leads to an increase in the PTS, but they performed a retrospective study of patients who had only undergone open-wedge HTO. El-Azab et al. [11] provided a possible explanation for the slope reduction after closed-wedge HTO, suggesting that the removal of a wedge from the anterolateral part of proximal tibia results in more bone loss at the anterior aspect since the wedge is not excised strictly laterally and perpendicular to the anatomical axis. However, many previous studies only considered standard hinge positions; they did not evaluate the effects of different hinge positions (standard or low). We sought to determine whether different hinge positions PTS. In this prospectively designed cadaveric study, we found that a low hinge position is significantly associated with a greater increase of PTS compared to the standard hinge position in medial open-wedge HTO while there were no significant differences in the closed-wedge HTO group. There are several reports on parameters related to open-wedge and closed-wedge HTO, but there are few simulated comparative studies of how different hinge positions impact outcomes. In this study, assessment of parameters for open-wedge and closed-wedge HTO in terms of hinge position could be considered a significant strength of this study.

There are several limitations of this work. First, because this is a cadaveric study, there were a limited number of subjects. Therefore, other factors such as the corrective angle and fixation plate choice were not considered. We sought to minimize limitations by selecting the optimal corrective angle and surgical protocols based on previously studied HTO outcomes [23, 24]. Second, evaluation of varus/valgus using long leg radiograph was impossible, given the nature of the design as it is impossible to place it in a standing, weight-bearing position was not plausible in a cadaveric study, and a preoperative long leg view was not attainable as it involved post-mortem joint contracture. Our primary goal was to observe changes in the PTS in HTO for different hinge positions. When selecting subjects, we tried to include normal knees without history of operation, knee pathology, or gross deformity. Third, the results were yield based on aged cadaveric knees, so factors such as bone mineral density (BMD) and bone morphometric changes may differ from those who are indicated for HTO, which may suggest our findings are not applicable to actual patients in such aspect. However, changes following osteotomy and intraoperative complications related to BMD, such as metal failure or subsidence, did not occur. Furthermore, because we used match-paired cadaveric knees to minimize the effects of other independent factors such as side-to-side difference, we assumed BMD did not affect our results. Bone morphometrics did not differ between the two groups in this study, but morphometrics for patients in the age range for which HTO is generally indicated could not be evaluated. Finally, because this was a cadaveric study, our study included only time-zero effects on knee alignment. It could not consider the effects during different postoperative periods. Some clinical studies including a retrospective analysis of patients following HTO with CT report that although scrupulous preoperative planning for ideal hinge position preceded HTO, the postoperative hinge position varied from the originally planned position[14, 27]. Compared to such reports, this study has an advantage because the random hinge positions were adopted so that the MPTA and PTS changes could be precisely analyzed in cadaveric knees. The clinical significance of PTS and MPTA changes during HTO has been widely reported, and long-term prognosis partially may even depend on hinge position. Therefore, establishing an accurate hinge position is a crucial factor for good prognosis.

## Conclusion

Tibial osteotomy at low hinge position should be avoided more in open-wedge than in closed-wedge HTO. Because this position increases the PTS and MPTA, there is a greater risk of lateral tibial cortex hinge fracture. Understanding the relationships between hinge position, PTS, and MPTA is critical for surgeons when performing open-wedge rather than closed-wedge HTO.

## Data Availability

Not applicable.

## Abbreviations

HTO: High tibial osteotomy
PTS: Posterior tibial slope
MPTA: Medial proximal tibial angle
OA: Osteoarthritis
UKA: Unicompartmental knee arthroplasty
ACL: Anterior cruciate ligament
PCL: Posterior cruciate ligament
BMI: Body mass index
BMD: Bone mineral density
